# Boosting clerkship preparedness: student insights on the effectiveness of an intensive post-COVID transition course

**DOI:** 10.1186/s12909-025-07479-y

**Published:** 2025-07-01

**Authors:** Quang Thanh Nguyen, Thuy Minh Ha, Siaw Cheok Liew, Jeffrey Mayne, Thuong Thi Ha Luong, Phuoc Van Le

**Affiliations:** 1 College of Health Sciences, VinUniversity, Hanoi, Vietnam; 2 Department of Pediatric Surgery, The National Hospital of Pediatrics, Hanoi, Vietnam

**Keywords:** Transition, Clerkship, Student’s perspectives, Course evaluation, Quality improvement, Curriculum design

## Abstract

**Background:**

The Medical Doctor program at VinUniversity, developed in collaboration with the Perelman School of Medicine at the University of Pennsylvania, comprises three years of preclinical education followed by three years of clinical rotations. The COVID-19 pandemic (2020–2023) disrupted medical education in Vietnam, underscoring the need for innovative strategies to address gaps in practical skills. In response, the Transition to Clinical Training (TCT) course was introduced in September 2023 to better prepare students for clinical clerkships.

**Methods:**

This study employed a mixed-methods approach to evaluate the effectiveness of the TCT course for first time implementation. Fourth-year medical students at VinUniversity participated in two surveys: the first immediately after completing the TCT course and the second at the end of their first clerkship year. The initial survey assessed students’ satisfaction with course content, organization, and teaching quality, while the follow-up survey focused on the course’s effectiveness in preparing students for clinical rotations. Quantitative data were collected using a 5-point Likert scale, and qualitative feedback was analyzed thematically.

**Results:**

The initial survey (88% response rate) indicated high satisfaction with the course’s content, organization, and teaching quality, though concerns were raised about grading fairness. The end-of-year survey (90% response rate) revealed moderate preparedness for clinical rotations, with specific challenges noted in interpreting laboratory tests and understanding hospital workflows. Qualitative feedback highlighted the need for increased hands-on practice, improved organization, and consistency in instruction.

**Conclusion:**

The TCT course at VinUniversity has substantially enhanced student readiness for clinical rotations, with strong satisfaction reported in several areas. However, there are critical areas for improvement, including grading fairness, laboratory test interpretation, and familiarity with hospital workflows. Ongoing refinement based on student feedback is essential to optimize the course and ensure comprehensive clinical preparedness.

**Supplementary Information:**

The online version contains supplementary material available at 10.1186/s12909-025-07479-y.

## Introduction

The Medical Doctor program in Vietnam is a six-year undergraduate course overseen by the Ministry of Health and the Ministry of Education and Training [1]. Established in 2020, VinUniversity’s College of Health Sciences undergraduate medical education (UME) program was developed in collaboration with the Perelman School of Medicine at the University of Pennsylvania (Upenn), and admitted its first class of medical students in 2020 [2]. This program includes three years of preclinical education followed by three years of hospital rotations, encompassing strategic planning, organizational structure, programmatic framework, curriculum design, lecture creation, and student evaluation [2, 3]. The theoretical components of the curriculum are primarily delivered in English, transitioning to a bilingual approach during hospital-based clinical rotations. The curriculum is designed to equip students with the necessary competencies to become proficient physicians either in Vietnam or internationally, preparing them for standardized exams such as the United States Medical Licensing Examination.

The COVID-19 pandemic caused substantial disruptions to education in Vietnam, severely impacting students from 2020 to 2023 [4]. During the early stages of the pandemic in 2020, the Vietnamese government enforced strict social distancing measures and lockdowns, resulting in the temporary closure of educational institutions. These measures led to significant interruptions, with schools and universities reopening intermittently based on local pandemic protocols [5]. VinUniversity’s first cohort of medical students, the class of 2020–2026, faced continuous disruptions throughout their preclinical years due to these social distancing measures. The prolonged nature of these disruptions underscored the urgent need for innovative solutions, such as intensive transition courses, to compensate for the practical aspects of education that were significantly hindered by the pandemic.

To address these challenges, the Transition to Clinical Training (TCT) courses w developed to equip students with the necessary skills and confidence to enter the clinical setting [6]. VinUniversity offered its inaugural TCT course in September 2023, one month before the clerkships began. This course aimed to bridge the gap in knowledge and practical skills caused by COVID-19 disruptions while also preparing students for the crucial transition from preclinical to clinical training. This transition is vital in medical education, requiring students to apply theoretical knowledge in real-world settings, handle increased responsibilities, and adapt to clinical environments.

This paper evaluates the TCT course at VinUniversity, integrating both quantitative and qualitative survey data from students. The study compares students’ perceptions and evaluations of the course through surveys conducted at the beginning and end of their clerkship year. Quantitative data were collected on course satisfaction, perceived learning outcomes, and clinical skills, while qualitative feedback provided deeper insights into students’ experiences and identified areas for improvement in the Transition to Clinical Training course.

## Method

### Design of Transition to Clinical Training (TCT) course

In the fourth year of the medical program at VinUniversity, students enter their first full-time clinical year (clerkship), which includes six core clinical rotations: Surgery (8 weeks), Internal Medicine (8 weeks), Pediatrics (4 weeks), Obstetrics & Gynecology (4 weeks), Neurology (4 weeks), and Psychiatry (4 weeks). Administrators at VinUniversity, in collaboration with adjunct clinical faculty from affiliated institutions, conducted a local needs assessment to determine the ideal learning outcomes for the course. As a result, a comprehensive 4-week program was designed to address both practical and theoretical aspects of clinical training, ensuring that students are well-prepared for the demands of their clinical clerkships. An in-person, multimodal teaching approach was adopted, incorporating lectures, case-based discussions, small group learning, clinical case presentations, simulated sessions with standardized patients, self-directed study, and hospital visits. The program covers three main components: Introduction to Clinical Medicine, Hospital Preparation, and Differential Diagnosis, as detailed in Table 1. The assessment methods were developed to align with the learning objectives and strategies are described in Table 2.

**Table 1:** Course outlines for the Transition to Clinical Training course

Components	Objectives	Topics Covered	Teaching Methods
Introduction to Clinical Medicine (ICM)	Develop fundamental clinical skills in history-taking, physical examination, and clinical reasoning.	• Principles of general examination • Head and neck examination • Cardiovascular interview and examination • Pulmonary and thoracic interview and examination • Abdominal interview and examination • Neurologic examination (including cranial nerves) • Introduction to Pediatrics • Introduction to Obstetrics and Gynecology • Introduction to Psychiatry • Mental Status Examination (MSE)	Lectures, simulated learning with standardized patients, small group learning, and self-directed learning.
Hospital Preparation (HP)	Familiarize students with the hospital environment, including workflows, protocols, and the use of electronic health records (EHR). Promote a quality and safe environment for patients and identify institutional expectations and appropriate behavior in the hospital.	• Hospital protocols • Patient safety • Organizational structure of patient care • Practical aspects of working in a hospital setting	Hospital visits, workshops on hospital protocols and EHR systems, and interactions with various healthcare teams.
Differential Diagnosis (DDX)	Enhance critical thinking and diagnostic reasoning skills. Develop differential diagnoses for common clinical presentations, formulate organized plans for initial evaluations, and recall diagnoses associated with multisystem diseases.	• Patient Presentation (Oral & Written) • Principles of Differential Diagnosis • Hypotension • Syncope • Musculoskeletal Pain • Constitutional Symptoms • Thoracic Symptoms • Gastrointestinal Symptoms • Gastrointestinal Bleeding • Jaundice • Alcoholism • Rash • Neurologic Symptoms • Neurologic Cases • Fever • Imaging Findings & Lab Findings • Multi-system Diseases	Lectures on common diagnostic challenges, case-based learning, small group discussions, and simulation exercises.

**Table 2:** Assessment plan for the Transition to Clinical Training course

Assessment Type	Description	Weighting
**Formative Assessments**	Continuous feedback from facilitators, peers, and standardized patients during simulation-based clinical and communication skills sessions, as well as small group case-based discussions.	-
**Summative Assessments**		
Group Work and Presentations	Each student participated in two group presentations, preparing and presenting clinical cases.	30%
Completion of Illness Scripts	Each student completed three individual illness scripts, including epidemiology, pathophysiology, clinical features, and differential diagnoses.	30%
Objective Structured Clinical Examination (OSCE)	OSCE with four stations assessing students’ abilities to conduct physical exams, patient assessments, and clinical procedures.	40%
**Professionalism**	Ongoing assessment of students’ professionalism, including participation, communication, and adherence to ethical standards.	Pass/Fail

All practical clinical simulations from the first to third year were reviewed, and the most relevant sessions for clerkship rotations were selected and thoroughly examined. The contents of each session were reconstructed with the collaboration of clinical faculty from the respective clerkship services. Following this revision, a comprehensive set of 10 clinical and communication sessions, known as Introduction to Clinical Medicine (ICM) sessions, was developed and is described in Table 1.

High-yield clinical topics, including differential diagnosis and case studies, were chosen to align with UPenn’s curriculum, with lecture materials also provided by UPenn. Emphasis was placed on contextual relevance, considering significant epidemiologic differences between American and Vietnamese populations. Consequently, a set of 23 diverse lectures was prepared, as detailed in Table 1. Lecturers were selected from actual training institutions where students will rotate during their clerkship. To enhance the quality of teaching of those newly recruited clinical faculty, faculty development sessions were conducted with the support of visiting faculty from UPenn.

During this time, each student spent three full days at one of the three affiliated hospitals where they would later rotate during their clerkship, allowing them to become familiar with that specific hospital’s workflows, protocols, and expectations. Teaching activities during hospital visits included shadowing exercises, where students followed hospital rounds, and interprofessional exercises, where students observed interactions between different healthcare teams. Students also had the opportunity to practice history-taking and physical examinations on actual patients under the guidance of the clinical faculty.

### Study design

This study aimed to investigate the research question from the students’ perspectives, using both qualitative and quantitative data to gauge the extent and depth of responses. Two surveys were conducted among fourth-year medical students at VinUniversity who had completed the TCT course.

- The first survey was administered in September 2023, immediately after the completion of the 4-week TCT course and just before the start of the clerkship year. This survey aimed to gather information on overall satisfaction with the course content, organization, quality of teaching, teaching materials, and fairness of workload and grading.
- The second survey was conducted in June 2024, near the end of the first clerkship year, to allow students to reflect on how well the TCT course prepared them for their clinical rotations. Feedback was collected on various parameters, including overall preparedness for clinical rotations, confidence in performing patient history and physical examinations, interpreting laboratory and diagnostic tests, communicating with patients and their families, collaborating with healthcare teams, utilizing electronic health records, and understanding hospital workflows and protocols. The questionnaires used for the survey were specifically designed to address the research question and are provided in the Supplementary Document.

Both surveys included quantitative questions rated on a 5-point Likert scale and open-ended qualitative questions. The quantitative questions assessed students’ ratings of the parameters, while the open-ended questions explored the most beneficial aspects of the TCT course and areas needing improvement. The complete questionnaire was reviewed for relevance and comprehensiveness. Before conducting the research, the self-designed evaluation questionnaire was administered to 10 randomly selected university students for pilot testing to ensure validity and reliability. All research instruments, including the consent forms and questionnaires, were available in English, as it is the language of instruction and assessment at VinUniversity.

### Data collection and analysis

The data were collected using a structured questionnaire specifically designed for this study and distributed via an electronic survey platform (Microsoft Forms). A research assistant helped disseminate the survey link to all fourth-year medical students following the completion of their didactic lectures. Participation was voluntary, and students submitted their responses online.

For the qualitative analysis, two authors (TMH and QTN), both experienced in qualitative research and clinical education, independently analyzed the open-ended responses using a qualitative descriptive approach. Each author applied descriptive coding to the responses, which were then grouped into subcategories and synthesized into broader themes. After the initial coding process, the researchers met to compare interpretations, discuss coding decisions, and ensure alignment with the research question. Any discrepancies were resolved through discussion, and when needed, a third author (PL) provided additional input to reach consensus. This collaborative process enhanced the consistency and analytical rigor of the thematic findings.

The data from the electronic survey were exported into an Excel spreadsheet (Microsoft Corporation, Redmond, WA) and statistical analysis was then performed using IBM SPSS Statistics version 25.0 for Windows. Descriptive statistics were presented as numbers, percentages, and mean ± standard deviation (SD). The average Likert scores for each item or factor were reported as mean ± SD. Significance levels were set at *p* < 0.05.

### Ethical considerations

This study received ethical approval from VinUniversity and the Ethical Committee of the Vinmec Healthcare System, registration number 55/2024/CN/HDDDVMEC. The questionnaire was accompanied by a cover letter that outlined the objectives of the study, emphasized that participation was entirely voluntary, detailed potential risks and benefits, and provided the authors’ contact information. Participants were encouraged to contact the researchers if they had any questions about the questionnaire. Informed consent was obtained from all participants before they took part in the study. To maintain confidentiality and anonymity, participants’ names and email addresses were removed upon form submission, and a unique ID was assigned to each respondent.

## Result

### Quantitative findings

The first survey was conducted upon completion of the TCT course and just before the onset of the clerkship year. Its primary objective was to collect feedback on the core learning objectives, structure, workload, and assessment of the course (Table 3). As students had not yet commenced their clerkship year, no questions were posed about the course’s effectiveness in preparing them for clerkship to avoid subjective and biased responses. Of the 48 students who completed the course, 42 responded to the survey, resulting in an 88% response rate.

**Table 3:** Quantitative data on course structure and effectiveness

Metric	Mean Score	Standard Deviation
**End of Course Evaluation**		
Overall Satisfaction with the course	4.24	0.76
Learning Objectives	4.48	0.59
Course Organization	4.07	0.89
Instructional Materials	4.21	0.72
Workload Fairness	4.07	0.71
Assessment Relevance	4.40	0.63
Grading Fairness	3.95	1.03
**End of Clerkship Reflection on Course Effectiveness**		
Overall Preparedness for Clinical Rotations	3.56	1.03
Performing History Taking and Physical Examinations	4.07	0.83
Interpreting Laboratory and Diagnostic Tests	3.14	1.13
Communicating with Patients and Their Families	3.91	0.95
Collaborating with Healthcare Teams	3.19	1.07
Utilizing Electronic Health Records	2.53	1.24
Understanding Hospital Workflows and Protocols	2.65	1.27

The results from the initial survey reflected a high level of satisfaction and perceived learning among students. The highest ratings were assigned to learning outcomes (mean score: 4.48) and assessment relevance (mean score: 4.40), indicating that students felt they had acquired significant knowledge and that the assessments were well aligned with the course content. However, the score for grading fairness was lower (mean score: 3.95), suggesting some concerns regarding the consistency and fairness of grading practices.

The second survey was conducted at the end of the first clerkship year, allowing students to retrospectively evaluate the effectiveness of the course after experiencing their clerkship. This survey aimed to gather insights into how well the course prepared them for clinical rotations. The response rate for the end-of-clerkship survey was 90%, with 43 out of 48 students participating. The findings are detailed in Table 3.

The results from the second survey indicated that while students felt prepared, there were notable areas for improvement. Specifically, the scores for interpreting laboratory and diagnostic tests (mean score: 3.14) and understanding hospital workflows and protocols (mean score: 2.65) highlighted these as particularly challenging areas.

### Qualitative findings

Twenty students (42%) submitted comments in response to the first survey, while 42 students (88%) provided comments in the second survey. The significant increase in responses to the open-ended questions at the end of the clerkship survey indicates the effectiveness of this follow-up survey. This increase is likely due to the increased amount of time at the end of the clerkship year and students having sufficient experience to reflect meaningfully on the preparatory course.

Selected examples representing frequently occurring themes in the comments are presented below under the relevant survey topic headings. The qualitative responses from the open-ended questions were categorized into key themes to highlight the most significant areas of feedback. This approach provides a clearer picture of student experiences and areas for improvement. For each theme, multiple representative quotes are included to illustrate the key points. The qualitative study data are summarized in Tables 4 and 5.

**Table 4:** Qualitative data collected at the end of Transitional to Clinical Training course

Themes	Description	Quotes
Hands-On Practice	Many students appreciated the practical approach but desired more hours dedicated to Introduction to Clinical Medicine (ICM) sessions. They emphasized the importance of hands-on practice in building their confidence and competence. Students expressed a need for more personalized feedback from instructors during these sessions.	*“More practice hours with ICM sessions would be helpful so that every student can receive feedback from instructors on their performance and how they could improve.”* *“The ICM sessions helped me a lot and I appreciate the feedback from instructors.”* *“Having more SPs (Standardized Patients) would be nice*,* especially for ICM sessions that require detailed physical examination practice.”*
Organizational Improvements	Better organization of study materials and earlier provision of syllabi were frequently suggested. Students felt this would help them better prepare for the intensity of the course. Having access to study materials ahead of time would allow students better understanding of course requirements and improved time management.	*“Syllabus and study materials could be given to students prior to the start of the course so that we could prepare our knowledge and mental state for the intensity of the course.”* *“I expect more from study materials like slides to help guide me through the course.”* *“Providing the course materials earlier would help us to come prepared and get the most out of each session.”*
Consistency in Instruction	Some students noted inconsistencies in examination technique of different instructors. This lack of standardization could lead to confusion and affect performance in assessment. Concerns were raised about how these inconsistencies could impact their grades, highlighting the need for clear and consistent teaching practices.	*“There are some inconsistencies in the clinical practices of instructors that we would like to make sure do not affect our grades in the examination.”* *“The consistency on assessment criteria among instructors. I think they should do simulation and have a mutual agreement on the correct techniques they want students to achieve.”* *“Different teaching styles and expectations can make it difficult to know how to prepare for exams.”*
Support and Feedback	Students appreciated the support and feedback from instructors, which helped improve their understanding and clinical skills. The availability and approachability of instructors were highlighted as a positive aspect of the course.	*“The instructors were very supportive and provided valuable feedback.”* *“I felt well-supported by the teaching staff*,* and their feedback was instrumental in my learning.”* *“The feedback after group presentations and during ICM sessions was very helpful in improving my skills.”*

**Table 5:** Qualitative data collected at the end of the first clerkship year

Themes	Description	Quotes
Early Integration and Extended Duration	Students recommended offering the TCT course earlier in the curriculum and extending its duration for more in-depth learning and practice. Early integration would allow for a more gradual buildup of clinical skills. Extending the duration of the course would provide students with more time to assimilate the material and practice their skills, thereby reducing the intensity and stress associated with a condensed timeframe.	*“Offering the course earlier in year 3 would provide a longer and more interactive experience.”* *“The course should run longer to allow for more practical time.”* *“Starting the course earlier in the program would help us build skills more gradually and effectively.”*
Increased Interactivity and Hospital Visits	Enhancing the interactivity of the course and incorporating more hospital visit days and surgical skills training were common suggestions. These elements were considered crucial for real-world clinical experience. Students valued the hands-on experience and the opportunity to apply their skills in practical settings, which reinforced their learning and increased their confidence.	*"Hospital visits are excellent; we got to learn and experience much more than previous years.”* *“The hospital visits were more demanding in terms of skills*,* but we got a more realistic insight and practice.”* *“More interactive sessions and real patient interactions would greatly enhance our learning experience.”*
Application of Skills and Knowledge	Students expressed the need for more opportunities to apply their theoretical knowledge in practical settings. They felt that real-world application was crucial for solidifying their learning. There were calls for more case-based learning and simulation exercises to enhance practical skills.	*“More opportunities to apply our knowledge in real-world settings would be very beneficial.”* *“Case-based learning and simulations helped me understand how to use my theoretical knowledge in practice.”* *“We need more practical sessions where we can apply what we’ve learned in a controlled environment.”*

## Discussion

The COVID-19 pandemic revealed the fragility of traditional medical education systems in the face of global crises. The rapid transition to virtual learning posed significant challenges while also fostering opportunities for innovation in medical education [7]. In 2021, Esquivel et al. developed a novel course aimed at bridging the gap between classroom learning and clinical practice during the COVID-19 disruption [8]. This course provided meaningful, skill-building clinical experiences in a supervised setting, developed through an iterative process involving all stakeholders. Various initiatives have emerged in the literature to mitigate the pandemic’s impact on medical education, although these initiatives vary significantly in structure and content and often miss high-yield topics essential for daily clinical practice [8, 9]. To address these shortcomings, VinUniversity implemented the Transition to Clinical Training (TCT) course, which emphasized simulation-based learning and practical workshops. These methods have proven effective in enhancing student preparedness for clinical rotations [10, 11].

Transitioning from preclinical education to clinical clerkships presents significant challenges even without the pandemic [12]. Many medical schools’ preclinical curricula lack emphasis on practical skills such as history-taking, physical examinations, oral presentations, documentation, and team dynamics [8, 12]. A survey by O’Brien et al. involving 83 medical schools found that while 73 offered some form of a transitional course, only 14 included clinical experiences [12, 13]. Less than one-third adequately covered essential skills like conducting a history and physical examination, interpreting laboratory data, or using basic medical equipment. Additionally, only about half addressed note-writing, order entry, and oral presentations [13].

A study by Atherley et al. highlights that limited clinical exposure leads to high anxiety and low self-confidence in clinical skills among preclinical medical students [14]. To address this, VinUniversity’s program dedicated three full days to hospital preparation. During these sessions, students visited affiliated medical institutions where they would complete their clerkships, allowing them to familiarize themselves with hospital workflows, protocols, institutional expectations, and appropriate behavior. Activities included shadowing hospital rounds and participating in interprofessional exercises, where students observed interactions between different healthcare teams. They also practiced history-taking and physical examinations on actual patients under the guidance of clinical faculty.

Aligning faculty and student expectations regarding clinical skills preparation is crucial, as discrepancies can lead to anxiety and reduced preparedness [15, 16]. To mitigate this, the TCT course involved clinical faculty from the training institutions where students would rotate during their clerkships. This early connection helped students understand expectations, significantly reducing their anxiety.

The evaluation of the TCT course among fourth-year medical students provided valuable insights into the program’s effectiveness in preparing students for clerkships. Data from surveys conducted at the beginning and end of the clerkship year, along with qualitative feedback, offered a comprehensive understanding of students’ experiences and identified areas for improvement.

### Key findings and implications from the study

1. **Moderate Improvement in Preparedness**: Scores indicated a moderate level of preparedness for clinical rotations, highlighting a gap between initial expectations and actual readiness. Addressing this gap is crucial for enhancing the course’s effectiveness. Ensuring students feel adequately prepared for clinical rotations will increase their confidence and reduce anxiety.
2. **Confidence in Clinical Skills**: High confidence in performing patient history and physical examinations (mean score: 4.07) demonstrates the effectiveness of practical training components. However, lower scores in interpreting laboratory and diagnostic tests (mean score: 3.14) emphasize the need for enhanced training in these areas. Focused workshops and additional practice opportunities could help bridge this gap.
3. **Effective Communication and Teamwork Training**: Moderate scores in communication with patients (mean score: 3.91) and collaboration with healthcare teams (mean score: 3.19) suggest that while some soft skills were developed, there is potential for further enhancement. Effective communication and teamwork are critical competencies in clinical settings, impacting patient outcomes and team dynamics. Continued emphasis on these skills is essential. Additional role-playing exercises, team-based projects, and communication workshops could further strengthen these areas.
4. **Proficiency in Electronic Health Records (EHR)**: The lower score for EHR utilization (mean score: 2.53) underscores the importance of integrating EHR training into medical curricula. Ensuring students are proficient in these systems prepares them for modern clinical practice and enhances their ability to deliver efficient and effective care. Practical sessions focused on EHR use, including simulated EHR environments, could improve competency in this area.

### Lessons learned and areas for improvement

1. **Increasing Hands-On Practice**: Both quantitative and qualitative data suggest that increasing hands-on practice hours, particularly in ICM sessions, could further enhance the course’s effectiveness. Personalized feedback from instructors during these sessions is crucial for student development. More opportunities for direct patient interaction and practice of clinical skills would be beneficial.
2. **Organizational Improvements**: Students suggested that better organization of study materials and earlier provision of syllabi would help them prepare more effectively for the course. Implementing these changes can enhance student readiness and reduce anxiety, allowing them to engage more fully with the course content. Streamlined communication and timely access to resources are key.
3. **Consistency in Instruction and Assessment**: Addressing inconsistencies in clinical practices taught by different instructors is essential for ensuring a standardized learning experience. Establishing clear guidelines and standardized assessment criteria promotes fairness and transparency, ensuring all students are evaluated consistently. Regular training and calibration sessions for instructors could help achieve this.
4. **Early Integration and Extended Duration**: Offering the TCT course earlier in the curriculum and extending its duration were common suggestions. This would allow students to have more time to assimilate the material and practice their skills, providing a more gradual transition to clinical practice. A longer course duration could also reduce the intensity and stress associated with a condensed timeframe.
5. **Increased Interactivity and Real-World Experiences**: Enhancing the interactivity of the course and incorporating more hospital visit days and surgical skills training were commonly suggested improvements. These elements are crucial for real-world clinical experience, providing students with the opportunity to apply their skills in practical settings. Incorporating more case-based learning, simulations, and interactive workshops could enhance the learning experience.
6. **Enhanced Instructor Support and Engagement**: While instructor support and feedback were generally praised, ensuring that all instructors are equally engaging and supportive could further improve student experience. Regular professional development for instructors on best teaching practices and student engagement strategies could be beneficial.

In summary, this study provides an insight evaluation of the effectiveness of a transition to clerkship courses, particularly for students whose education was severely impacted by COVID-19 disruptions. To our knowledge, this is the first study of its kind, employing a robust design that includes two surveys. Additional data collected at the end of the clerkship year, when students had sufficient experience to reflect meaningfully, offered more reliable and insightful findings. The results indicated moderate improvements in preparedness for clinical rotations, with notable gaps in areas such as interpreting laboratory data and using electronic health records. The study underscores the need for enhanced practical training and consistency in instruction to better prepare medical students for clinical practice.

The flexible structure of the TCT course, initially developed to address educational disruptions caused by COVID-19, aligns with the broader global shift toward competency-based, practice-oriented medical education [6]. By adapting international benchmarks to local contexts, particularly within low- and middle-income countries, this curriculum offers a practical and context-sensitive approach to medical training. Documenting and evaluating such an innovative course provides further insights into curriculum reform, contributing to ongoing discussions about how medical schools worldwide can effectively balance global standards with local educational needs and healthcare realities.

### Limitations

This study offers a quantitative and qualitative analysis of a transition course from the perspectives of medical students. However, there are several limitations to consider. One primary limitation is the relatively small sample size, which restricts the generalizability of the findings to other settings or larger populations.

Additionally, the study relies heavily on self-reported data, which introduces the potential for response bias. Participants may have consciously or unconsciously reported their experiences and perceptions in a manner that reflects more favorably on the course’s effectiveness. This bias can affect the validity of the findings.

Another key limitation of this study is the absence of a pre-existing version of the Transition to Clinical Training course for comparison. As VinUniversity’s medical program is relatively new, with the first cohort admitted in 2020 and the inaugural TCT course introduced in 2023, there is no historical control group or baseline data from a prior curriculum model. This limits our ability to assess the relative effectiveness of the TCT course over time.

Future research should address current limitations by including larger and more diverse student samples such as additional cohorts or multi-institutional participation to improve the generalizability of findings. Incorporating objective measures of clinical performance and patient outcomes would also provide further evidence of the TCT course’s long-term effectiveness in preparing students for clinical practice. Additionally, gathering perspectives from clinical faculty involved in clerkship supervision could offer complementary insights into how the course has influenced student readiness and performance in real-world settings, enriching the evaluation with a broader, practice-based viewpoint.

## Conclusion

The Transition to Clinical Training course at VinUniversity has proven to be an effective program for bridging the gap between preclinical education and clinical practice. This comprehensive evaluation, integrating student feedback and survey data, demonstrates that the TCT course significantly enhances students’ preparedness for their clerkship rotations. High ratings in various aspects, including clinical skills, diagnostic reasoning, communication, and teamwork, highlight the course’s strengths. By continuously evolving and integrating student feedback, the TCT course at VinUniversity can ensure that future medical professionals are well-equipped to face the demands of clinical practice, even amidst unprecedented challenges caused by COVID-.

## Electronic supplementary material

Below is the link to the electronic supplementary material.

Supplementary Material 1

## Data Availability

The datasets used and/or analysed during the current study are available from the corresponding author on reasonable request.

## Abbreviations

DDX: Differential Diagnosis
EHR: Electronic Health Record
ICM: Introduction to Clinical Medicine
OSCE: Objective Structured Clinical Examination
SP: Standardized Patient
TCT: Transition to Clinical Training
UME: Undergraduate Medical Education
UPenn: University of Pennsylvania
