# Supplementary material for: Boosting clerkship preparedness: student insights on the effectiveness of an intensive post-COVID transition course
Source: BMC Med Educ. 2025 Jul 1;25:967. doi: 10.1186/s12909-025-07479-y (PMC12220478; doi:10.1186/s12909-025-07479-y)
Supplement: Supplementary file 1 — Supplementary Material 1 [file 12909_2025_7479_MOESM1_ESM.docx]

**QUESTIONNAIRE: Evaluation of the Transition to Clinical Training (TCT) Course: Assessing Immediate Impact and Long-Term Preparedness for Clinical Clerkship**

**Part I: Transitional to Clinical Training Course. End of Course Evaluation Survey.**

**Post-Course Evaluation of the Transition to Clinical Training (TCT) Course: Immediate Feedback Survey**

Thank you for participating in the evaluation of the Transition to Clinical Training (TCT) course. Your feedback is valuable in helping us improve the course for future students. The purpose of this survey is to assess the overall quality, structure, and effectiveness of the TCT course in preparing you for clinical rotations.

Please rate each statement based on your experience with the TCT course. Use the scale provided to indicate your level of agreement with each statement. Your responses will remain anonymous and will be used solely for the purpose of course improvement.

Rating Scale:

1 = Strongly Disagree

2 = Disagree

3 = Neutral

4 = Agree

5 = Strongly Agree

**Please rate the following statements:**

1. I am satisfied with the overall quality of the Transition to Clinical Training (TCT) course.
2. The learning objectives of the TCT course were clear and aligned with my expectations.
3. The TCT course was well-organized and structured effectively.
4. The instructional materials provided (e.g., slides, notes) were helpful and relevant to the course content.
5. The workload of the TCT course was fair and manageable.
6. The assessments were relevant to the course content and effectively evaluated my understanding.
7. The grading system for the TCT course was fair and transparent.

**COMMENT SECTION:**

1. **What specific aspects of the course or instructor(s) were particularly helpful in supporting your learning?**

……………………………………………………………………………………………………

1. **Are there any specific areas of the course that could be improved to better support your learning?**

……………………………………………………………………………………………………

1. **Do you have any other comments regarding your overall learning experience in this course?**

…..………………………………………………………………………………………………..

**Part II. Evaluation of the Transition to Clinical Training (TCT) Course: End of Clerkship Year Reflection.**

**End-of-Clerkship Evaluation of the Transition to Clinical Training (TCT) Course: Student Reflection Survey**

Thank you for taking the time to participate in this survey. The purpose of this study is to evaluate the effectiveness of the Transition to Clinical Training (TCT) course, which you completed at the start of your clerkship year, in preparing you for real-world clinical practice. By reflecting on your experiences during your clerkship, your feedback will help us improve the course for future students and ensure it continues to meet the needs of medical trainees.

This survey is part of an ongoing research study aimed at enhancing medical education at VinUniversity. Your responses will remain completely confidential and anonymous, and no identifying information will be collected. The data will be used solely for the purpose of improving the TCT course and related educational initiatives.

**Instructions:**
Please rate how much you agree with the following statements based on your experiences during the clerkship year. Use the following scale:

1 = Strongly Disagree
2 = Disagree
3 = Neutral
4 = Agree
5 = Strongly Agree

**Looking back on the Transition to Clinical Training course you completed at the start of your clerkship year, please rate how much you agree with the following statements regarding how the course prepared you for your clerkship:**

1. The course effectively prepared me for performing patient history and physical examinations.
2. The course effectively prepared me for interpreting laboratory and diagnostic tests.
3. The course effectively prepared me for communicating with patients and their families.
4. The course effectively prepared me for collaborating with healthcare teams.
5. The course effectively prepared me for utilizing electronic health records.
6. The course effectively prepared me for understanding hospital workflows and protocols.

**COMMENT SECTION:**

1. **What aspects of the Transition to Clinical Training course did you find most beneficial?** (Please describe the components of the course that best supported your preparation for clinical practice.)

……………………………………………………………………………………………

1. **What aspects of the course do you think need improvement?**
   (Are there specific areas where the course could be enhanced to better support your learning and preparation for clerkship?)

……………………………………………………………………………………………

1. **Is there anything else you would like to add?**
   (Please provide any additional feedback or reflections on your experience with the TCT course and its impact on your clinical training.)

……………………………………………………………………………………………
